# Synergies Between Observed Warming and ENSO Episodes on Extreme Events

**DOI:** 10.1111/nyas.70122

**Published:** 2025-11-04

**Authors:** Francisco Estrada, Pierre Perron, Yohei Yamamoto

**Affiliations:** ^1^ Instituto De Ciencias de La Atmósfera y Cambio Climático Universidad Nacional Autónoma De México, Ciudad Universitaria Circuito Exterior Mexico; ^2^ Institute for Environmental Studies Vrije Universiteit Amsterdam Netherlands; ^3^ Programa De Investigación en Cambio Climático Universidad Nacional Autónoma De México, Ciudad Universitaria Circuito Exterior Mexico; ^4^ Department of Economics Boston University Boston Massachusetts USA; ^5^ Department of Economics Hitotsubashi University Tokyo Japan; ^6^ Academy of Energy and Informatics Institute of Science Tokyo Tokyo Japan

**Keywords:** attribution, climate change, El Niño/Southern Oscillation (ENSO), generalized extreme value, risks on natural and human systems, temperature and precipitation extremes

## Abstract

El Niño/Southern Oscillation (ENSO) is the dominant interannual variability mode of the global climate system with significant effects on a variety of weather conditions, including extremes. Past events illustrate the severe societal consequences this phenomenon has through weather disasters, food security, health, economic growth, migration, and conflict. ENSO's interactions with global warming are not well understood, although they can lead to significant changes in the characteristics of extreme events. Climate conditions in 2024/2025 may favor widespread severe extreme events with global temperature anomalies nearing or surpassing 1.5°C and a transition from strong El Niño to La Niña conditions. Here, we show that current warming has amplified the effects of ENSO on temperature and precipitation extremes worldwide. Results show that warming has produced a considerable amplification of the effects of ENSO episodes over such extremes, as well as extensively modified spatial patterns. We show that considerable shares of the population, gross domestic product, agriculture, and ecosystems now face a higher risk from extreme events due to the interactions between increased anthropogenic forcing and ENSO.

## Graphical abstract

The literature has shown that anthropogenic climate change has affected the probabilities of occurrence of extremes around the world. However, the synergies between natural variability oscillations and current warming over extremes have been seldom studied. Here, we analyze how the observed levels of anthropogenic radiative forcing have changed the spatial patterns and magnitudes of probabilities of occurrence of extreme temperatures and precipitation events when strong ENSO episodes occur. The graphical abstract shows the change in probabilities of exceeding the 90^th^ percentile in maximum temperatures due to the interaction between a strong El Niño episode and current warming.

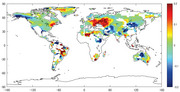

## 1 Introduction

Several natural variability modes have climate‐related effects over world regions [1]. El Niño/Southern Oscillation (ENSO) is the largest source of interannual variability of the climate system with widespread effects on temperature and precipitation patterns, including extreme events [2, 3, 4, 5, 6, 7, 8]. ENSO is an ocean−atmosphere coupled oscillation with an irregular 2‐ to 7‐year cycle having climatic and societal effects [1, 9]. It has three phases: neutral, El Niño, and La Niña, corresponding to near‐zero, positive, and negative sea surface temperature (SST) anomalies in the eastern equatorial Pacific, respectively. They affect trade winds, the slope of the thermocline, and upwelling in the tropical Pacific [3, 9, 10]. They also generate changes in the atmospheric convection, modifying the Walker circulation and producing shifts in the intertropical convergence zone [1, 3].

Many studies show how the characteristics of climate extremes are changing under a warming climate, as well as the associated thermodynamic and dynamic mechanisms [11, 12, 13, 14, 15, 16, 17, 18, 19]. Both long‐term and event attribution studies evidence the influence of anthropogenic emissions [11, 20, 21, 22, 23, 24, 25] and the need for enhanced climate policy and compensation schemes for loss and damage [26, 27, 28, 29]. The effects of climate change on ENSO led to mixed conclusions. The evidence shows a 20% change in its amplitude during the late 20th century [30]. The frequency of strong ENSO episodes has increased since the 1960s [31], and anthropogenic forcing signals are present in various characteristics of ENSO [3, 30, 32].

Studies documented the impacts of ENSO on natural and human systems via different channels [8, 33], including extreme floods, droughts [34], wildfires [35], storms and tropical cyclones [36, 37, 38], crop yield reductions [39, 40], fisheries and marine ecosystems [41], diseases and health problems [42]. The global impacts of recent extreme El Niño events (1997–1998, 2015–2016) are in the range of $2–4 trillion, while those from La Niña are asymmetric and considerably smaller [43]. In present value, the economic impact of increased ENSO variability caused by climate change is valued at $33 trillion [43]. Some argued that the interaction between natural variability and increased total radiative forcing (TRF) increased such economic damages [44].

Our analysis is based on generalized extreme value (GEV) models estimated per grid cell with time‐invariant shape and scale parameters and time‐varying location parameters. The latter is a function of TRF, and some of the main natural variability modes like the Southern Oscillation Index (SOI), the Atlantic Multidecadal Oscillation (AMO), the North Atlantic Oscillation (NAO), and the Pacific Decadal Oscillation (PDO), as well as stratospheric aerosols (VOL), are also included as control variables. Here, we discuss results with no explicit interaction terms between ENSO and TRF, while the supplement presents results with this interaction term. We create counterfactual scenarios, namely, the difference in the effect of a plus/minus 2 standard deviation in ENSO under the current levels of forcing and preindustrial conditions, with TRF set to NAT, the natural component of the forcing, and at zero for AMO, NAO, PDO, and VOL [11]. The results reported are the differences in the exceedance probabilities in temperature and precipitation extremes between these two scenarios for strong El Niño/La Niña events. The increased risk refers to the increased probabilities of exceeding the 90th quantile of the observed distribution of the 3‐month maximum over the period 1961–1990 induced by a change in ENSO when setting TRF to its current level compared to preindustrial levels. The main results in this paper emphasize the atmospheric component of ENSO by using SOI to represent ENSO, and we provide complementary results based on the SST‐focused Niño‐3.4. The literature has recognized the existence of various flavors of ENSO and that they can have substantially different impacts on the global climate [6, 31, 45]. The present manuscript focuses exclusively on the canonical (Eastern Pacific) ENSO; other ENSO variations are out of scope but flagged for future work.

We show that the risks of extreme temperatures/precipitation due to the interaction effects between TRF and strong ENSO events have increased steeply for sensitive areas with high population and/or high gross domestic product (GDP), as well as in areas with high biodiversity and agricultural value. Since TRF is still increasing, the interaction effects between rising radiative forcing and ENSO phases will strengthen, suggesting that the system's response will be increasingly amplified [46, 47].

## 2 Methods

### 2.1 Data Sources and Processing

The extreme temperature and precipitation indices TXx and RX1day were obtained from the Hadley Centre's HadEX3 dataset [19] (available at https://www.metoffice.gov.uk/hadobs/hadex3/), which has a global land coverage with a spatial resolution of 1.875° longitude and 1.25° latitude. Data for these variables are available for 1901–2018. Both variables were interpolated to match the 0.5° × 0.5° spatial resolution of the SSP5 population and GDP projections included in the CLIMRISK integrated assessment model [27, 48, 49]. These are consistent with the SSP5 scenario [49] and the estimates used pertain to population and GDP (US$2005) in the year 2018 (available at https://datapincc.unam.mx/datapincc/#).

The effective radiative forcing (ERF) used for our models is that of Ref. [50], which follows the methodology used in the IPCC's Sixth Assessment Report (AR6) [51] and covers the period 1750–2022 (https://github.com/ClimateIndicator/forcing‐timeseries/tree/main/output). The ERF components are aggregated as total, anthropogenic, and natural, as in Ref. [50]. Here, we define the TRF as the sum of all anthropogenic (ANT) and natural (NAT) radiative forcings, with the exception of the forcing from stratospheric aerosols (VOL). VOL is included in the analysis as a separate variable, as it can distort the trend in the aggregated radiative forcing [52].

The analysis presented here focuses on the canonical Eastern‐Pacific ENSO, while other flavors [45], like the central Pacific (Modoki) and the coastal El Niño, are beyond the scope of the paper and left for future work. As discussed in the next section, several indices have been developed to represent ENSO conditions. Here, we select the SOI to represent ENSO in the main model specification and the ENSO3.4 index for a complementary analysis contained in the Supporting Information. SOI and El Niño‐3.4 monthly anomalies are moderately correlated (*r* ≈ 0.57), as expected for coupled ocean–atmosphere variability, but they emphasize different physical processes (sea‐level pressure vs. sea‐surface temperature). SOI provides a more direct representation of the atmospheric component of this coupled phenomenon, and it is expected to accentuate the circulation pathways in our analysis. The ENSO3.4 index by construction provides a better representation of ENSO's oceanic component and thus highlights thermodynamic moisture‐energy pathways. The SOI time series was obtained from the Climate Research Unit (CRU; available at https://crudata.uea.ac.uk/cru/data//soi/soi.dat), and the ENSO3.4 index was obtained from https://climexp.knmi.nl/getindices.cgi?WMO=NCDCData/ersst_nino3.4a_rel&STATION=NINO3.4_rel&TYPE=i&id=ff918db159c510a5f55cd67fd8a9925e. Although we refrain here from analyzing other ENSO flavors and additional ENSO indices (e.g., the Multivariate ENSO Index, the Oceanic El Niño Index), we recognize these as valuable extensions beyond this paper's scope.

Given the reported interplay between the AMO [53, 54, 55], PDO [56, 57], NAO [58, 59], and ENSO, these variability modes are included in the analysis, and their effects on modulating the ENSO impacts on extreme temperature and precipitation are accounted for. These time series were obtained from https://climexp.knmi.nl/getindices.cgi?STATION=AMO_ersst&TYPE=i&WMO=NCDCData/amo_ersst&id=$id (AMO), https://crudata.uea.ac.uk/cru/data/nao/nao_3dp.dat (NAO), and https://www.ncei.noaa.gov/pub/data/cmb/ersst/v5/index/ersst.v5.pdo.dat (PDO). These variability modes cover annual to decadal timescales and are used to account for high to low frequencies that could distort the effects of ENSO and TRF over the dependent variable—in this case, extreme temperature and precipitation [11, 52, 60]. We treat these indices as physically motivated controls rather than causal targets. The Arctic Oscillation (AO) and the North Pacific Oscillation (NPO) indices were used to conduct a sensitivity analysis of the group of control variables selected for this study. These variables were obtained from https://climatedataguide.ucar.edu/sites/default/files/2024‐04/npindex_monthly.txt for NPO, and https://climexp.knmi.nl/dat2nc.cgi?datafile=data/iao_slp_ext.dat&type=i&station=AO_SLP for AO.

In addition to AMO, NAO, and PDO, other indices such as the AO, NPO, and Arctic Sea ice anomalies could provide regionally specific insights. However, we prioritize a parsimonious specification that includes NAO, AMO, and PDO as they represent the dominant modes of low‐frequency variability and avoid potential redundancies [61, 62]. For instance, NAO captures the regional expression of the hemispheric variability of AO, while NPO's signal is largely embedded in PDO phases. Including overlapping indices risks multicollinearity and overfitting, particularly given the limited observational record of extremes. Furthermore, other variables, such as Arctic sea ice anomalies, while increasingly relevant for midlatitude extremes [13], were excluded as their effects are indirectly mediated by NAO/AMO in our model. Sensitivity tests in the Supporting Information confirm that results are robust when including AO and NPO.

### 2.2 GEV Time Series Models

Our analysis is based on GEV models estimated per grid cell with time‐invariant shape and scale parameters and time‐varying location parameters. Such models have been used before to separate the contributions of anthropogenic and natural radiative forcing to changes in exceedance probabilities and return levels on extreme temperature and precipitation variables [11]. As detailed below, the location parameter is a function of the TRF, including greenhouse gases, anthropogenic aerosols, and natural forcing factors (solar and stratospheric aerosols); SOI, AMO, NAO, and PDO are also included as control variables. In addition, we consider two specifications of the model: (a) no explicit interaction terms between the natural variability modes and TRF; (b) with the inclusion of interaction terms between radiative forcing and SOI. Model (b) attempts in some crude way to account for nonlinear effects. However, given the extra number of parameters involved, it may be prone to overfitting biases. The results between the two models are qualitatively similar but much stronger with model (b). The main text presents the conservative results from model (a) and those of model (b) are included in the Supporting Information.

There are several indices for characterizing ENSO, which emphasize either the atmospheric or oceanic components of this coupled phenomenon [63]. Two widely used examples are the SOI and the ENSO3.4 index [64, 65, 66]. The SOI quantifies sea‐level pressure differences between Darwin (Australia) and Tahiti (central Pacific), directly reflecting atmospheric dynamics such as shifts in the Walker Circulation. In contrast, ENSO3.4 represents detrended SST anomalies averaged over the central Pacific (5°N–5°S, 170°–120°W), capturing oceanic thermal forcing. Here, we prioritize SOI to isolate atmospheric teleconnections, which are critical for mediating precipitation extremes and modulating temperature responses via circulation changes (e.g., convection shifts and Rossby wave propagation). However, in the Supporting Information, we provide complementary results for model (a) using ENSO3.4 to explore how SST‐driven thermal forcing interacts with TRF and contributes to regional extremes. This dual approach underscores ENSO's coupled nature while clarifying the distinct roles of atmospheric and oceanic drivers under anthropogenic warming. Let $x_{i,t}$ be the maxima of daily temperature/precipitation over a specific 3‐month period (December–January–February for winter and June–July–August for summer) in year t and geographical grid i. We specify the distribution of $x_{i,t}$ as being generated by the GEV distribution, namely, $x_{i,t}∼GEV\left(\mu_{i,t},\sigma_{i},\xi_{i}\right)$ with

(1) $$\begin{array}{ccc} \mu_{i,t} & = & \mu_{0,i}+\mu_{1,i}TRF_{t}+\sum_{j=0}^{2}\delta_{i,\left[j\right]}^{+}SOI_{t,\left[j\right]}^{+}+\sum_{j=0}^{2}\delta_{i,\left[j\right]}^{-}SOI_{t,\left[j\right]}^{-}\\ & & +\;\mu_{2,i}NAO_{t}+\mu_{3,i}AMO_{t}+\mu_{4,i}PDO_{t}+\mu_{5,i}VOL_{t}\\ & & +\;\sum_{j=0}^{2}\gamma_{i,\left[j\right]}^{+}\left(TRF_{t}\times SOI_{t,\left[j\right]}^{+}\right)+\sum_{j=0}^{2}\gamma_{i,\left[j\right]}^{-}\left(TRF_{t}\times SOI_{t,\left[j\right]}^{-}\right). \end{array}$$

In Equation (1), $TRF_{t}$ is the annual TRF (minus radiative forcing caused by stratospheric aerosols $VOL_{t}$) measured in year t. $SOI_{t,[j]}^{+}$ is the jth lag of the 3‐month average of the SOI in year t if it has a nonnegative value and zero otherwise. For example, when we consider December–January–February, $SOI_{t,[0]}^{+}$ is the average of December–January–February in year t, $SOI_{t,[1]}^{+}$ is the average of September–October–November in year t−1, and $SOI_{t,[2]}^{+}$ is the average of June–July–August in year t−1. $SOI_{t,[j]}^{-}$ is similarly defined if the SOI in year t has a negative value. $NAO_{t}$, $AMO_{t}$, $PDO_{t}$ are the 3‐month averages of the NAO Index, AMO Index, and PDO Index in year t, respectively. $VOL_{t}$ is the 3‐month average of the volcanic radiative forcing in year t. The coefficients associated with these variables as well as $\mu_{0,i}$, $\sigma_{i}$, and $\xi_{i}$ are estimated by the method of maximum likelihood using annual data from 1901 to 2018 for each geographical grid i. Denote the estimates of these parameters by using a hat, such as ${\hat{\mu }}_{0,i}$. Model (a), which is used for the estimations presented here, imposes no explicit interactions between TRF and ENSO by restricting the parameters $\gamma_{i,[j]}^{+}$ and $\gamma_{i,[j]}^{-}$ to be zero. Model (b) relaxes such restrictions and the results are presented in Figures S3 and S4. The preference for model (a) is due to its parsimony. Model (b) requires the estimation of several additional parameters, potentially leading to overfitting problems, and lower reliability and performance. In particular, overfitting can make parameters more variable and less stable, and lead to the under‐ and overestimation of risk.

Note that the parameters of interest are $\mu_{0,i},\mu_{1,i}$, $\delta_{i,[j]}^{+},$ $\delta_{i,[j]}^{-}$ for model (a); while for model (b), in addition to $\gamma_{i,[j]}^{+}$ and $\gamma_{i,[j]}^{-}$. The coefficients on $NAO_{t},AMO_{t},$ $PDO_{t}$, and $VOL_{t}\;$ will play no role, but are needed to obtain the estimates of the parameters of interest, since it is important to control for the modulating impact of these various natural variations.

These models are used to create two sets of counterfactual scenarios, namely (1) under the current levels of forcing and (2) preindustrial conditions. Each set of scenarios depicts the effects of a strong ENSO episode on temperature and precipitation extremes under neutral phases of AMO, NAO, PDO, and VOL. The differences in the exceedance probabilities in temperature and precipitation extremes between these two sets of scenarios are compared to quantify the joint effects of increased forcing and El Niño/La Niña phases. The probability that $x_{i,t}$ exceeds the 90 percentile of the 3‐month maximum over the period 1961–1990, denoted by $q_{i}$, can be computed via the following formula:

(2) $$\mathrm{Pr}\left(x_{i,t}\geq q_{i}\right)=1-exp\left\{-\left[1+{\hat{\xi }}_{i}{\left(\frac{q_{i}-{\hat{\mu }}_{i}}{{\hat{\sigma }}_{i}}\right)}^{-\frac{1}{{\hat{\xi }}_{i}}}\right]\right\},$$

where

(3) $$\begin{array}{ccc} {\hat{\mu }}_{i,t} & = & {\hat{\mu }}_{0,i}+{\hat{\mu }}_{1,i}{\hat{TRF}}_{t}+\sum_{j=0}^{2}{\hat{\delta }}_{i,\left[j\right]}^{+}{\hat{SOI}}_{t,\left[j\right]}^{+}+\sum_{j=0}^{2}{\hat{\delta }}_{i,\left[j\right]}^{-}{\hat{SOI}}_{t,\left[j\right]}^{-}\\ & & +\;{\hat{\mu }}_{2,i}{\hat{NAO}}_{t}+{\hat{\mu }}_{3,i}{\hat{AMO}}_{t}+{\hat{\mu }}_{4,i}{\hat{PDO}}_{t}+{\hat{\mu }}_{5,i}{\hat{VOL}}_{t}\\ & & +\;\sum_{j=0}^{2}{\hat{\gamma }}_{i,\left[j\right]}^{+}\left({\hat{TRF}}_{t}\times {\hat{SOI}}_{t,\left[j\right]}^{+}\right)+\sum_{j=0}^{2}{\hat{\gamma }}_{i,\left[j\right]}^{-}\left({\hat{TRF}}_{t}\times {\hat{SOI}}_{t,\left[j\right]}^{-}\right), \end{array}$$

where the various variables with a “^” denote counterfactual values. We are interested in the probabilities of extreme events under the following scenarios. The first pertains to the current level of warming (radiative forcing) and the second to the preindustrial level of warming, for which we use the natural radiative forcing in 2018, denoted by $NAT_{2018}$. The effect of El Niño is assessed by the difference in probability computed by setting ${\hat{SOI}}_{t,[j]}^{-}=-2{\hat{\sigma }}_{SOI}$, where ${\hat{\sigma }}_{SOI}$ is the sample standard deviation of the historical monthly series of the SOI, and the effect of La Niña is computed by setting ${\hat{SOI}}_{t,[j]}^{+}=2{\hat{\sigma }}_{SOI}$. We assume a long‐term equilibrium scenario for the various natural modulating factors by setting ${\hat{NAO}}_{t}={\hat{AMO}}_{t}={\hat{PDO}}_{t}={\hat{VOL}}_{t}=0$. Then, the El Niño effect under the first scenario is

(4) $$\begin{array}{ccc} \pi_{cur,ElNi\tilde{n}o} & = & \mathrm{Pr}\left(x_{i,t}\geq q_{i}|{\hat{TRF}}_{t}=TRF_{2018},{\hat{SOI}}_{t,\left[j\right]}^{-}=-2{\hat{\sigma }}_{SOI}\right)\\ & & -\;\mathrm{Pr}(x_{i,t}\geq q_{i}|{\hat{TRF}}_{t}=TRF_{2018},{\hat{SOI}}_{t,\left[j\right]}^{-}=0), \end{array}$$

with ${\hat{SOI}}_{t,[j]}^{+}=0$.

The La Niña effect under the first scenario is computed as:

(5) $$\begin{array}{ccc} \pi_{cur,LaNi\tilde{n}a} & = & \mathrm{Pr}\left(x_{i,t}\geq q_{i}|{\hat{TRF}}_{t}=TRF_{2018},{\hat{SOI}}_{t,\left[j\right]}^{+}=2{\hat{\sigma }}_{SOI}\right)\\ & & -\;\mathrm{Pr}(x_{i,t}\geq q_{i}|\;{\hat{TRF}}_{t}=TRF_{2018},{\hat{SOI}}_{t,\left[j\right]}^{+}=0), \end{array}$$

with ${\hat{SOI}}_{t,[j]}^{-}=0$.

These scenarios allow us to isolate the effects of El Niño/La Niña, plus their interaction with $TRF_{2018}$, from the direct effects of $TRF_{2018}$.

For the second scenario, the El Niño effect is computed as:

(6) $$\begin{array}{ccc} \pi_{pre,ElNi\tilde{n}o} & = & \mathrm{Pr}\left(x_{i,t}\geq q_{i}|{\hat{TRF}}_{t}=NAT_{2018},{\hat{SOI}}_{t,\left[j\right]}^{-}=-2{\hat{\sigma }}_{SOI}\right)\\ & & -\;\mathrm{Pr}(x_{i,t}\geq q_{i}|\;{\hat{TRF}}_{t}=NAT_{2018},{\hat{SOI}}_{t,\left[j\right]}^{-}=0), \end{array}$$

with ${\hat{SOI}}_{t,[j]}^{+}=0$; and the La Niña effect is

(7) $$\begin{array}{ccc} \pi_{pre,LaNi\tilde{n}a} & = & \mathrm{Pr}\left(x_{i,t}\geq q_{i}|{\hat{TRF}}_{t}=NAT_{2018},{\hat{SOI}}_{t,\left[j\right]}^{+}=2{\hat{\sigma }}_{SOI}\right)\\ & & -\;\mathrm{Pr}(x_{i,t}\geq q_{i}|\;{\hat{TRF}}_{t}=NAT_{2018},{\hat{SOI}}_{t,\left[j\right]}^{+}=0). \end{array}$$

These scenarios isolate the El Niño/La Niña, plus the interaction effects with $NAT_{2018}$, from the direct effects of $NAT_{2018}$.

The main results reported are the differences between the counterfactuals concerning the differential effect of a 2‐sigma deviation of El Niño/La Niña under a preindustrial level of TRF, in which case, ${\hat{TRF}}_{t}=NAT_{2018}\;$, and the effect of a similar deviation under the current level of TRF, in which case, ${\hat{TRF}}_{t}=TRF_{2018}\;.$ These are computed as $\lambda_{i,ElNi\tilde{n}o}=\pi_{cur,ElNi\tilde{n}o}\;-\pi_{pre,ElNi\tilde{n}o}$ for a strong El Niño event and for a strong La Niña event as $\lambda_{i,LaNi\tilde{n}a}=\pi_{cur,LaNi\tilde{n}a}\;-\pi_{pre,LaNi\tilde{n}a}$. These are the desired measures of the increase in the probability of exceedance due exclusively to the interaction effects of the different phases of ENSO and the increases in the (mostly) anthropogenic increase in radiative forcing, that is, $TRF_{2018}$ relative to $NAT_{2018}.$ Note that they refer exclusively to how probabilities of exceeding the 90th quantile of the observed distribution of the 3‐month maximum over the period 1961–1990 have changed due to these interaction effects, not to the marginal effects of increases in TRF or variations in ENSO.

To assess the statistical significance of SOI, likelihood ratio tests were conducted for each geographical grid during the DJF and JJA seasons:

For El Niño, the current and lag effects of the negative SOI are zero.

(8) $$H_{0}:\;\;\delta_{i,\left[0\right]}^{-}=\delta_{i,\left[1\right]}^{-}=\delta_{i,\left[2\right]}^{-}=0.$$

$$H_{1}:\;\mathrm{At}\;\mathrm{least}\;\mathrm{one}\;\mathrm{of}\;\mathrm{the}\;\mathrm{equations}\;\mathrm{in}\;H_{0}\;\mathrm{does}\;\mathrm{not}\;\mathrm{hold}.$$

For La Niña, the current and lag effects of the positive SOI are zero

(9) $$H_{0}:\;\;\delta_{i,\left[0\right]}^{+}=\delta_{i,\left[1\right]}^{+}=\delta_{i,\left[2\right]}^{+}=0.$$

$$H_{1}:\;\mathrm{At}\;\mathrm{least}\;\mathrm{one}\;\mathrm{of}\;\mathrm{the}\;\mathrm{equations}\;\mathrm{in}\;H_{0}\;\mathrm{does}\;\mathrm{not}\;\mathrm{hold}.$$

The significance test results are presented in Figure S1 for extreme temperature and Figure S2 for extreme precipitation.

## 3 Results

### 3.1 Changes in Risk for Precipitation and Temperatures

It has been previously shown that anthropogenic drivers increased the probabilities of exceedance in temperatures/precipitation up to fivefold/threefold with respect to 1961–1990. About 94%/72% of the global population and 97%/76% of the global GDP now face a higher risk [11]. These changes in probabilities of occurrence and spatial patterns are expected to be modified during the different phases of ENSO [3, 30]. While both global warming and ENSO individually drive extreme events through thermodynamic (e.g., moisture amplification) and dynamic (e.g., jet stream shifts) mechanisms [13, 67, 68, 69], our analysis isolates their nonlinear interaction effects. First‐order effects (e.g., ENSO's classic teleconnections, TRF's baseline warming) are statistically removed in $\lambda_{i,ElNi\tilde{n}o}$ and $\lambda_{i,LaNi\tilde{n}a}$ to focus exclusively on synergies. The main mechanisms for the estimated changes in probabilities of exceedance are: nonlinear amplification of ENSO's impact on extremes due to increases in higher background warming and higher moisture‐holding capacity of the atmosphere; changes in intensity and duration of ENSO episodes; shifts in traditional teleconnection patterns; and possible generation of emergent dynamics and pathways [30, 70, 71].

At present, although the literature describing the joint effects of ENSO and global warming is vast [3, 30, 70, 71, 72], studies focusing on higher‐order effects between ENSO and TRF are not abundant, particularly concerning extreme events [30, 73, 74, 75, 76], and usually based on climate models’ output. Here, we provide an observational‐based analysis to show the importance of the TRF−ENSO interactions and to describe them. However, while we offer below some potential physical drivers for the observed higher‐order effects, describing in detail the specific physical processes and mechanisms that may generate them is beyond the scope of this work. The main contribution of the present paper is to analyze whether these interaction effects between ENSO and TRF are already quantifiable in the data and to estimate how they may have modified the probability of occurrence and spatial patterns of extremes during the observed period.

**FIGURE 1:**
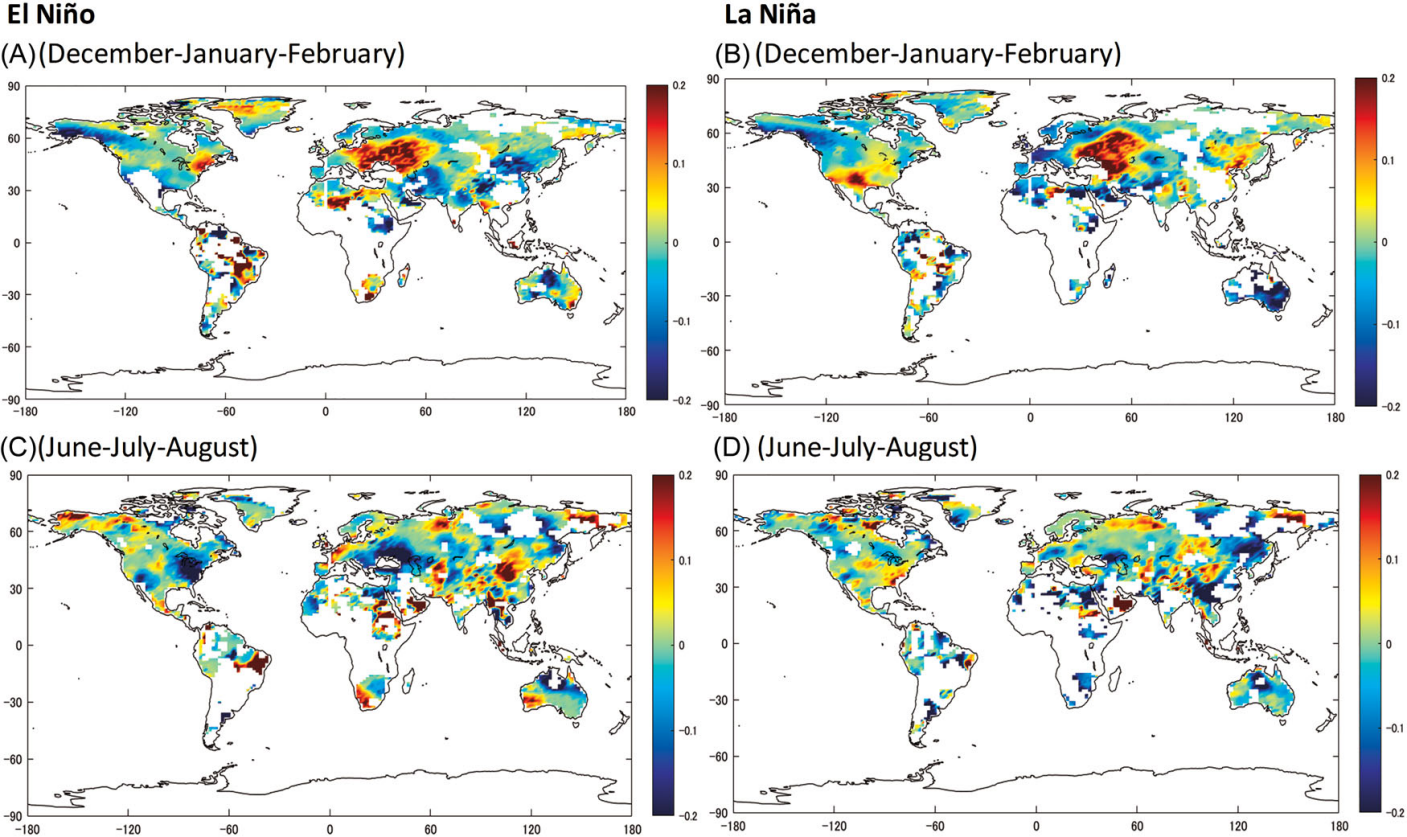
Synergistic impacts of strong El Niño/Southern Oscillation (ENSO) events and total radiative forcing (TRF) on the probability of extreme temperature. A strong El Niño is defined as a scenario in which the Southern Oscillation Index (SOI) continues to be −2 standard deviations of the historical SOI series (1901:1–2018:12) over the current and the past two quarters consecutively. A strong La Niña is defined similarly, but the SOI index is +2 standard deviations. The synergistic impact of these events and TRF is assessed across each geographical grid by calculating the effect of a strong ENSO event on the estimated probability of exceeding the extreme threshold under the 2018 TRF level and that calculated under a preindustrial TRF level. Here, the threshold is set at the 90th percentile of the highest daily temperatures in the 3‐month period of a calendar year from 1961 to 1990. The synergistic effects in the left panels (A and C) are denoted by $\lambda_{i,ElNi\tilde{n}o}\;$ and those in the right panels (B and D) are denoted by $\lambda_{i,LaNi\tilde{n}a}$ as detailed in Methods.

Figure 1 shows the changes in temperature risk produced by the effects of the interactions between anthropogenic forcing and strong positive/negative phases of ENSO for boreal winter (upper panel) and summer (lower panel). Increases in TRF have substantially changed the probabilities of temperature risk during strong ENSO episodes across seasons and the globe. Due to their interactions, in the northern hemisphere, high temperature risk in winter during a strong El Niño event (Figure 1A) has increased 20% or more in Eastern Europe and Russia, parts of Algeria, Niger, and Libya, when compared to a similarly strong El Niño event under preindustrial forcing. The US East Coast and Quebec also show increases in temperature risk near 15%−20%, as well as Bangladesh and Indonesia. Decreases near 20% occurred in the Middle East and central Asia, particularly in the UAE, Saudi Arabia, Eastern China, and Mongolia. In America, such decreases during winter occurred in Alaska, the Yukon and Northwest territories in Canada, and the north of the Gulf of Mexico coast. In Ethiopia and Sudan, temperature risk in winter has decreased by up to 20%. These results can be partially due to the nonlinear amplification of ENSO's effects over extreme events. For example, enhanced warming due to persistent high‐pressure systems product of Rossby wave resonance, as well as reduced cloud cover and rainfall, induced by a weakened Walker circulation due to anthropogenic intervention [73], which can amplify temperature extremes already increased by climate change. In the southern hemisphere, the results in Figure 1A pertain to the summer. Then, the ENSO−TRF interactions induced increased temperature risk greater than 20% over regions of Brazil and Colombia, as well as in South Africa, and with a smaller magnitude near the Canberra and Sidney areas in Australia. Large decreases in risk are present in northern Brazil, Paraguay, parts of Bolivia, Venezuela, and Chile, and the central part of Australia. In some of these regions, El Niño episodes can bring reductions in cloud cover and relative humidity that can increase the probability of extreme temperature risk. These changes can be amplified by, for example, northward shifts in the Intertropical Convergence Zone caused by differential warming between hemispheres [77, 78]. Other higher‐order effects between TRF and El Niño that can modify temperature risks involve changes in surface energy balance and soil−atmosphere interactions [79].

For a strong La Niña episode (Figure 1B, winter), the synergistic effects lead to considerably larger temperature risk (>20%) in parts of Russia and central Asia, the southern US, and northern México. The increase reaches 20% in northern China and Libya. However, there is a moderate to large decrease over central Europe, the Middle East, northern Africa, Alaska, and northwest Canada in winter. This is also the case during summer for South America (except parts of Brazil, Chile, and Bolivia) and Australia. The largest decreases in risk occur in Australia, parts of Brazil and Colombia, Egypt, and eastern Saudi Arabia. In parts of China, interactions between background warming due to climate change and the local/regional effects of urban warming [80] contribute to the amplification of temperature risks during La Niña episodes. In some regions, the interactions between TRF and La Niña modify temperature risks through direct increases in background temperature, changes in atmospheric circulation, primarily the Walker circulation and jet streams, and in pressure systems, cold air advection, as well as in soil−atmosphere interactions [1, 73, 81, 82]. During the boreal summer (Figure 1C), we observe decreases in temperature risk greater than 20% for most of central Asia, the Balkans, west Africa, a significant part of eastern US and Canada, the southwest of the US, and northern Mexico. In the case of the Balkans and Central Asia, TRF−ENSO interactions can lead to the weakening of the subtropical jet stream and shifts in storm tracks, which can increase precipitation and cloud cover, boosting the release of latent heat and reducing solar insolation [83, 84, 85]. Under anthropogenic warming, these conditions may be further enhanced due to nonlinear amplification processes such as intensified convective and latent heat feedbacks, as well as an increased sensitivity of atmospheric circulation to SST anomalies in a warmer and moister climate. In the southern hemisphere's winter, substantial decreases are present in northern Australia. In contrast, large increases occur in the northwest of Brazil, southeast Australia, and Africa during winter, and during summer in Alaska and Mexico, central and western Europe, southeast of the Middle East, Bangladesh, and Myanmar, as well as northern China. A strong La Niña episode (Figure 1D) induces moderate increases (10%−15%) in temperature risk in the summer over large parts of Asia, the US east coast, Mexico, the Netherlands, and Spain, while large increases (>20%) occur mainly in parts of the Middle East, eastern US, Russia, northern China, and Mongolia. The largest reductions in temperature risk are in southern China, southeastern Russia, central and eastern parts of North Africa, parts of northern Brazil, and Argentina. These spatially heterogeneous changes in risk may arise from the modulation of classical ENSO teleconnections due to climate change, including changes in the mean state of the Pacific, the position and strength of the Walker circulation, and higher‐order interactions between ENSO and other warming‐sensitive variability modes. As in the case of boreal winter, the effects of TRF and La Niña in boreal summer produce a small but generalized decrease in temperature risk in the southern hemisphere, possibly reflecting changes in extratropical feedbacks and enhanced ocean−land interactions under warmer conditions.

**FIGURE 2:**
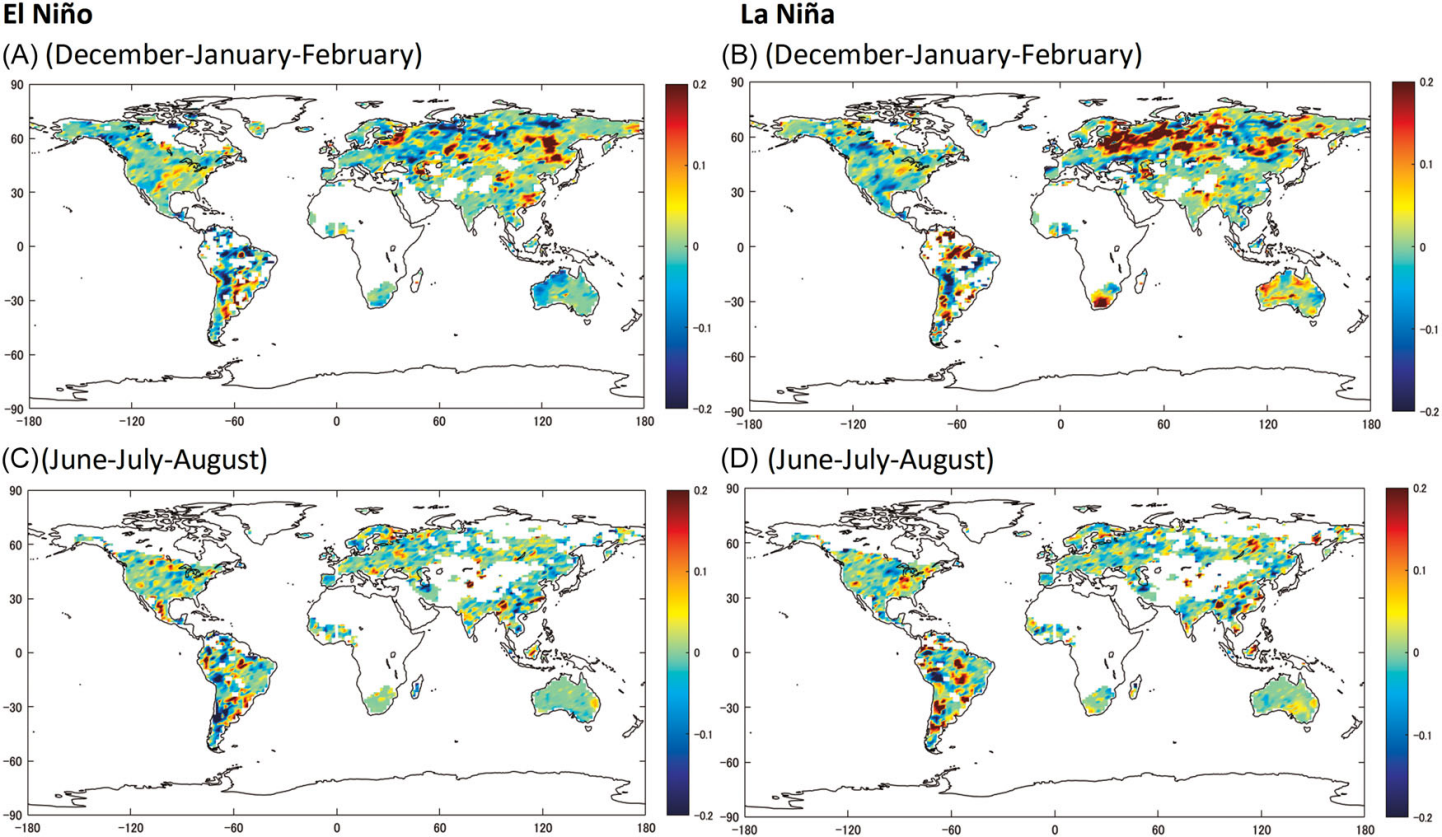
Synergistic impacts of strong El Niño/Southern Oscillation (ENSO) events and total radiative forcing (TRF) on the probability of extreme precipitation. A strong El Niño is defined as a scenario in which the Southern Oscillation Index (SOI) remains to be −2 standard deviations of the historical SOI series (1901:1–2018:12) over the current and the past two quarters consecutively. A strong La Niña is defined similarly, but the SOI index is +2 standard deviations. The synergistic impact of these events and the TRF is assessed across each geographical grid by calculating the effect of a strong ENSO event on the estimated probability of exceeding the extreme threshold under the 2018 TRF level and that calculated under a preindustrial TRF level. Here, the threshold is set at the 90th percentile of the largest daily precipitations in the 3‐month period of a calendar year from 1961 to 1990. The synergistic effects in the left panels (A and C) are denoted by $\lambda_{i,ElNi\tilde{n}o}\;$ and those in the right panels (B and D) are denoted by $\lambda_{i,LaNi\tilde{n}a}$ as detailed in Methods.

Consider now the effects of ENSO phases and increases in TRF for precipitation (Figure 2). They are especially pronounced in both summer and winter for South America and in boreal winter for high and mid latitude regions in Eastern Europe and Asia. The changes in risk in boreal winter are larger and more spatially homogenous for a strong La Niña episode, with increased risk of extreme precipitation in eastern Europe, Russia, and Australia. Global warming can further enhance these effects, as the increased atmospheric moisture content and enhanced latent heat feedback from warmer SSTs could intensify storm activity. The effects of a strong El Niño are less pronounced and mirror those of La Niña, namely, decreased risk in these regions. In summer, the ENSO/TRF interaction has no clear impact on extreme precipitation in Europe and Asia. With a strong El Niño episode in summer, precipitation risks increase notably over most of Mexico which could be linked to enhanced atmospheric moisture content and convective sensitivity in the region due to warming. In South America, considerable changes in risk occur during both strong El Niño and La Niña phases and across seasons. For most of northern South America, the TRF−El Niño interactions lead to reduced precipitation risk, and those of TRF−La Niña to increases. In contrast, the southwest of South America is associated with decreased risk, and the southeast with an increase. These complex and regionally contrasting patterns may be influenced by nonlinear feedbacks, including the intensification of latent heat release and SST‐convection coupling, and possible emergent interactions between ENSO and other modes of variability under a warmer and more humid background climate.

As shown in Figure S1, for temperature risk, the effect of El Niño in the boreal summer and La Niña in the boreal winter is particularly significant in Latin America, South and East Asia, and Australia. The effect is relatively less significant in Eastern Europe, as well as in Central Asia, that is, the mid‐to‐high latitudes of Eurasia and Africa (where the data is available). For extreme precipitation risk (Figure S2) in the boreal winter, ENSO's impact is more significant in Latin America, eastern Australia, and Mexico. However, similar to temperature risk, the effect is less significant in Eastern Europe and in Central Asia.

Figures S3 and S4 show results for the alternative specification in which an interaction term between ENSO and TRF is explicitly included as a variable. The changes for both temperature and precipitation risk are more widespread and much larger. It is not straightforward to assess which model specification provides a better representation of the overall effects. However, the main qualitative result remains the same: the interactions between TRF and ENSO have changed the levels of risk and their spatial patterns for both temperatures and precipitation. In addition, Figures S5 and S6 provide a sensitivity analysis exploring the differences in changes in temperature and precipitation risks that are obtained from using the ENSO3.4 index instead of SOI. As mentioned in Methods, the ENSO3.4 index may be better at representing the interactions that are more closely related to oceanic thermal forcing. While, in general, the results using ENSO3.4 are broadly similar to those of SOI, there are considerable regional differences for both temperature and precipitation risk, particularly for Eastern Europe and Central Asia during a strong La Niña episode in winter. The Supporting Information also contains a sensitivity analysis that shows results are broadly similar when including AO and NPO in the group of control variables (Figures S7 and S8).

### 3.2 Changes in Risks for Human and Natural Systems

Studies documented large ENSO effects on societies and natural systems globally [35, 39, 40, 43]. Here, we assess the additional risks ENSO−TRF interactions pose to population and GDP (Table 1), as well as in vulnerable systems such as agriculture and biodiversity (Table 2).

**TABLE 1:** Synergistic effects of strong El Niño/Southern Oscillation (ENSO) events and total radiative forcing (TRF) on the probability of occurrence of extreme temperature and precipitation events over global/regional population and gross domestic product (GDP).

	El Niño‐TRF		El Niño‐TRF		La Niña‐TRF		La Niña‐TRF	
	Winter		Summer		Winter		Summer	
Population	TXx	RX1day	TXx	RX1day	TXx	RX1day	TXx	RX1day
All world	27.5	11.1	25.5	14.4	23.0	12.5	17.4	15.6
US	21.2	17.6	2.3	12.1	38.6	11.6	29.0	16.3
Western Europe	32.8	11.9	42.7	7.1	11.5	3.6	31.0	8.5
Japan	25.0	8.0	53.8	0.0	11.6	6.7	0.0	0.0
Russia	70.9	17.4	3.4	14.9	57.3	57.9	2.7	6.7
Eastern Europe and Central Asia	62.5	15.6	17.2	13.6	39.2	16.7	30.8	8.0
China	16.5	23.3	20.4	11.1	42.4	5.2	3.4	24.9
India	14.4	0.9	18.6	14.8	15.5	22.0	20.8	11.0
Western Asia	16.6	1.3	41.2	0.0	53.1	4.0	5.7	0.2
Africa	26.2	10.4	27.8	11.7	4.9	11.4	15.7	14.7
Latin America (exc. Mexico)	53.3	15.1	29.6	25.2	14.7	16.7	14.6	34.4
Canada, Australia, NZ, Korea, Taiwan	29.0	17.6	1.9	11.6	8.0	1.6	0.0	14.8
Other Asia	38.8	3.5	32.6	22.0	12.7	9.5	26.6	11.0
Mexico	1.6	0.7	65.9	36.4	4.1	12.1	11.1	5.9

	El Niño‐TRF		El Niño‐TRF		La Niña‐TRF		La Niña‐TRF	
	Winter		Summer		Winter		Summer	
GDP	TXx	RX1day	TXx	RX1day	TXx	RX1day	TXx	RX1day
All world	26.8	12.1	26.9	12.5	25.0	9.7	17.5	15.0
US	21.8	17.6	2.2	11.9	39.0	11.8	28.6	16.1
Western Europe	15.8	5.2	58.0	6.9	2.3	1.9	29.7	6.8
Japan	25.7	7.8	54.1	0.0	11.7	6.7	0.0	0.0
Russia	71.2	17.2	3.3	15.0	56.7	58.1	2.5	6.6
Eastern Europe and Central Asia	72.3	12.1	10.6	14.3	27.6	19.8	32.2	9.2
China	15.3	22.9	18.9	10.9	45.5	4.9	2.6	24.6
India	15.6	0.7	17.6	14.4	15.3	21.9	19.4	11.3
Western Asia	20.4	1.0	42.4	0.0	55.4	2.4	10.4	0.2
Africa	26.4	3.7	18.3	7.6	10.9	13.7	5.9	8.9
Latin America (exc. Mexico)	64.9	15.0	25.7	27.2	20.9	13.5	12.4	36.3
Canada, Australia, NZ, Korea, Taiwan	38.7	11.5	2.4	14.8	6.3	2.3	0.0	18.4
Other Asia	58.6	3.4	41.3	23.3	10.2	5.4	30.3	8.5
Mexico	1.4	0.8	67.5	36.1	4.2	12.6	10.4	5.1

*Note*: The numbers (in %) indicate the proportions of population and GDP exposed to an increase of more than 5% in the risk of extreme temperature (TXx) and precipitation (RX1day) to the total population and GDP in regions where the risk can be calculated.

**TABLE 2:** Synergistic effects of strong El Niño/Southern Oscillation (ENSO) events and total radiative forcing (TRF) on the probability of occurrence of extreme temperature and precipitation events over agricultural and high biodiversity areas.

Effects	El Niño‐TRF Winter	El Niño‐TRF Summer	La Niña‐TRF Winter	La Niña‐TRF Summer
Agricultural land				
Maize				
TXx	14.5 [30.6]	18.3 [26.4]	26.0 [32.8]	23.5 [27.3]
RX1day	17.4 [12.9]	17.2 [14.6]	10.2 [14.4]	18.3 [20.5]
Rice				
TXx	21.1 [32.9]	25.7 [29.7]	30.3 [29.1]	9.4 [16.6]
RX1day	19.6 [13.5]	17.5 [14.2]	12.8 [13.3]	22.4 [22.9]
Soybean				
TXx	19.6 [12.8]	12.4 [24.7]	26.8 [37.5]	25.1 [26.1]
RX1day	23.4 [24.0]	21.6 [13.7]	16.0 [13.3]	27.1 [21.9]
Wheat				
TXx	20.7 [24.4]	17.0 [16.6]	21.0 [23.3]	15.7 [13.6]
RX1day	13.1 [15.6]	14.1 [15.3]	11.3 [12.7]	13.2 [12.9]
High biodiversity land				
Mammals				
TXx	20.1 [19.6]	22.3 [22.4]	18.2 [18.1]	17.2 [17.5]
RX1day	15.0 [15.0]	13.2 [12.9]	22.9 [22.3]	16.6 [16.1]
Amphibians				
TXx	37.5 [27.9]	31.2 [40.4]	12.1 [4.8]	12.6 [13.1]
RX1days	15.9 [9.7]	21.4 [16.3]	37.0 [24.4]	30.0 [27.9]

*Note*: Numbers in the agricultural land section denote the percentage of grid cells with high yields (above average) in which the change in the probabilities of exceedance increases at least 5%, while those in brackets show similar percentages for low yield (below average) cells. Numbers in the high biodiversity land denote the percentage of grid cells with high species richness (above average) in which changes in the probabilities of exceedance increase at least 5%, while those in brackets show similar percentages for cells with high richness of endangered species. TXx, risk of extreme temperature. RX1day, risk of extreme precipitation.

A strong El Niño‐TRF event raises extreme temperature/precipitation risk by over 5% for about 27.5%/11.1% of the global population and 26.8%/12.1% for the global GDP. For a strong La Niña, these figures are 23.0%/12.5% in winter and 17.4%/15.6% in summer for the global population; for global GDP, they are 25.0%/9.7% in winter and 17.5%/15.0% in summer. Areas with high population densities (>5 million people per grid cell) experienced increased risks up to 70.9% in extreme temperature risk under a strong El Niño and up to 57.3% for La Niña. Regions with high economic exposure (>US$100 billion per grid cell) show similar changes in risk, with increases of 72.3% in winter under El Niño and up to 56.7% under a strong La Niña in summer.

Figures S9 and S10 show the hotspots of changes in risk for areas with high concentrations of population and GDP. In winter, a strong El Niño events increase temperature risks (Figure S9A) mostly (>15%) for large population centers in South America, the Balkans, Indonesia, and the Middle East. During the summer (Figure S9C), the hotspots of population with the largest increases in temperature risk are in the Philippines, north‐central Europe, Japan, Mexico, and southeast Asia, while those with the largest decreases are in South America, the Balkans, and Russia. The increases in precipitation risk are much smaller than for temperature in large population agglomerations (Figure S10A,C), with large parts having a reduction in risk, both in winter and summer, except for Argentina, Brazil, and the Balkans.

A strong La Niña episode in boreal winter implies higher temperature risk for large population centers in the Balkans, the southwest of Saudi Arabia, and California, and to some extent in Argentina, Chile, India, and China (Figure S9B). During the summer, there is a moderate but widespread increase in large urban areas in central Europe, especially the Balkans and Indonesia (Figure S9D). As in the case of El Niño, precipitation changes in risk are smaller compared to those of temperatures (Figure S10B,D). In winter, the hotspots with high increases in precipitation risk are mostly located in the northern hemisphere (Russia, northern US, central Mexico, India), while during the austral winter, they occur mostly in South America and China.

Figures S11 and S12 present the hotspots for changes in risk for high GDP exposure areas. A strong El Niño in summer produces the largest increases in temperature risk in central and northern Europe, Mexico, and Japan. The largest increases are in the southern hemisphere occur for the austral summer (Brazil, Argentina, Indonesia, and Chile). The effects of a strong La Niña in summer produce a moderate increase in temperature risk for most regions with high GDP exposure in Europe and North America. In contrast, during a La Niña episode in winter, areas in Europe experience a decrease in temperature risk, while high and moderate increases are present in many US regions. Both the interactions between El Niño/La Niña and TRF in winter produce moderately increased precipitation risks for regions with high GDP exposure in Europe and the US, as well as parts of China and Japan. For Mexico and Russia, increased TRF/strong La Niña also leads to increased risk. During the summer, higher precipitation risks occur in Quebec, Buenos Aires, and Shanghai.

Table 2 provides results for natural and human systems. Studies showed that extreme events have significant impacts on plant growth, crop yields, and the production of various staples [86, 87, 88, 89]. We document the portion of crop area devoted to maize, rice, soybean, and wheat with increased risk. These provide more than two‐thirds of the caloric energy in human diets and are central for global food security [90, 91, 92, 93, 94]. Results show that land devoted to rice production has the largest area under additional temperature risk. For highly productive areas, this occurs with a strong La Niña event in boreal winter, with 30.3% of the area having temperature risks higher than 5%. For low‐yield areas, the maximum area under increased temperature risk reaches 37.5% for soybean under a strong La Niña episode. The main conclusion is that increased TRF and a strong El Niño event generates increased temperature risk for about 12%−26% of high‐yield areas and 13%−33% of low‐yield areas. Under a strong La Niña event, the percentages for high‐yield areas are in the range of 9%−25% in summer, 21%−30% in winter, and for low‐yield areas 23%−38% in winter and 14%−27% in summer. For precipitation risk, a strong El Niño event implies increased risk for 13%−23% of high‐yield areas, and for La Niña, 10%−27%. For low‐yield areas, these figures are 13%−24% for El Niño and 13%−23% for La Niña.

Studies showed the significant consequences of climate extremes over biological systems [95, 96, 97], with some events having permanent effects [98]. A recent one argued that many land vertebrates would be exposed to extreme thermal events, with the geographical range exposure increasing this century [96]. Table 2 shows the percentage of high‐richness areas of mammals and amphibians facing increased temperature and precipitation risks (>5%) due to strong ENSO−TRF interactions. For mammals, the largest percentage of areas under additional temperature risk occurs during strong El Niño events, particularly in summer. For increased precipitation risk, this occurs for strong La Niña episodes in winter. The results are similar for all mammals and endangered species. Overall, the increase in areas with additional risks due to ENSO−TRF interactions is in the range 13%−23%. For amphibians, the largest additional areas with increased temperature risk occur during strong El Niño episodes (38% for total in winter and 40% in summer for endangered), while for precipitation, they are highest during strong winter La Niña episodes (37% total) and strong summer La Niña events (28% endangered). In general, the increase in TRF produces additional risks when a strong ENSO episode occurs for about 5%−40% of the areas with high and endangered biodiversity worldwide.

## 4 Discussion

Previous efforts analyzed the changes in the probabilities of exceedance of extreme events due to climate change and the contributions of natural/anthropogenic drivers, while others have done so for natural variability modes, such as ENSO. However, the extent to which their synergies modify the probability and spatial distribution of extremes has received comparatively less attention. This study provides observational evidence that interactions between TRF and ENSO have considerably amplified the risks of extreme temperature and precipitation events, even under conservative model specifications that exclude explicit interaction terms. These interactions have likely contributed to observed events. For example, the extreme precipitation and flooding in Rio Grande do Sul, Brazil, during April–May 2024 occurred in the context of a strong El Niño event and about 1.3°C of global temperature increase with respect to preindustrial conditions. An event‐attribution study suggests that both El Niño and anthropogenic warming substantially increased the likelihood and severity of the event [99]. According to our results (Figure 2C), nonlinear interactions between El Niño and TRF may have increased the probability of these types of events in the region by 20%. Similarly, the record‐breaking heatwave in Mexico during May–June 2024, which has also been linked to human‐induced warming and coincided with a strong El Niño phase [100], is consistent with our estimates of a 15% increase in the probability of extreme temperature extremes in parts of Mexico due to TRF/ENSO interactions (Figure 1C). While formally associating the changes in risk presented here to any single event requires dedicated studies, these examples are coherent with the estimated spatial and seasonal patterns of compound ENSO–TRF risk.

The amplification of extreme temperature and precipitation risk associated with ENSO–TRF interactions has material implications for human and natural systems. Our results show that significant portions of the global population, economic output, agricultural land, and biodiversity hotspots are already exposed to increased risk under current forcing conditions. For instance, up to 62.5% (72.3%) of areas with high population (GDP densities), particularly in Latin America, parts of Asia, and Europe, have seen increases in risk during strong ENSO episodes due to ENSO/TRF interactions. Similarly, staple crop regions and high‐richness biodiversity areas show widespread increases in risk from extremes, with potential implications for food security, livelihoods, and ecosystem stability. Doubling current levels of forcing, as some scenarios project for the end of this century (e.g., SSP370, SSP585), would make climate variability events much more difficult to manage and endure for human and natural systems.

To our knowledge, there is no prior global, observation‐based analysis that removes first‐order effects of both ENSO and the anthropogenic warming trend and focuses exclusively on the ENSO−TRF interaction for temperature and precipitation extremes. The closest observational work combines ENSO with trends regionally without isolating interaction effects [101], and most evidence for changing teleconnections is model‐based, underscoring the need for complementary empirical work. While detailed mechanism identification requires targeted process studies such as event composites, reanalysis diagnostics, and model experiments, which are outside this paper's scope, we offer a basic mechanistic framing that can help explain the synergies documented in our findings.

Several physical mechanisms plausibly underpin the state‐dependent changes in the probabilities of temperature and precipitation extremes arising from ENSO−TRF interactions. Thermodynamic intensification raises column water vapor and integrated vapor transport, while higher land vapor‐pressure deficit increases evapotranspirative demand [102, 103, 104]. Together, these factors amplify ENSO‐related wet extremes and compound hot–dry extremes [105, 106]. Dynamical modulation of the background state [107, 108, 109, 110] (e.g., weakening/shift of the Walker circulation, Hadley expansion, and latitude/strength changes in the subtropical and eddy‐driven jets) alters Rossby‐wave sources and pathways [111], shifting storm tracks [109], blocking, and atmospheric‐river landfalls [112], so that similar ENSO‐related SST anomalies project differently onto regional extremes. Convective sensitivity also increases: in warm/moist regimes, CAPE tends to rise and/or CIN to decrease, enhancing latent‐heating and upper‐level divergence, while in warm/dry regimes, drier boundary layers and higher CIN suppress local convection while strengthening the response to remote tropical heating, deepening teleconnection wave trains. Over land, stronger soil−moisture−temperature coupling and reduced snow/ice enhance sensible heating and boundary‐layer depth, intensifying hot‐dry ENSO anomalies. In addition, pattern dependence matters as changes in the tropical Pacific mean SST gradient and the relative occurrence of EP versus CP (Modoki) events reshape teleconnections [113], with decadal background modes (such as the PDO) further modulating the response. Collectively, these processes imply that ENSO's influence under warming is increasingly multiplicative rather than additive [76]. Moreover, emergent interactions between global warming and ENSO, as well as with other variability modes, may become more relevant as warming increases and affect midlatitude and tropical dynamics in complex ways. As TRF levels continue to increase, the higher‐order effects identified here are likely to become more prominent, underscoring the importance of considering the interactions between natural variability and anthropogenic change as potential amplifiers or modulators of climate extremes.

The interactions of anthropogenic forcing and natural variability have already changed the levels of risk and their spatial and seasonal distributions. Recent discussions have argued whether the climate conditions experienced since 2023 could be a sign of a fundamental change in the climate system or the expected effects of a strong El Niño event [114]. The observed amplification of the risk of extreme temperature and precipitation events due to TRF/ENSO interaction suggests increasing nonlinear responses and positive feedback mechanisms. Results presented here hint that the climate system response to natural variability is already different, even without any observable evidence of crossing any known tipping point yet [115].

## Author Contributions

F.E., P.P., and Y.Y. contributed equally to this work.

## Conflicts of Interest

The authors declare no competing interests.

## Code Availability Statement

The code for reproducing the results is available upon request.

## Supporting information




**Figure S1**: Significance of the ENSO effects on the probability of extreme temperature: Model (a).
**Figure S2**: Significance of the ENSO effects on the probability of extreme precipitation: Model (a).
**Figure S3**: Synergistic impacts of strong ENSO events and TRF on the probability of extreme temperature: Model (b).
**Figure S4**: Synergistic impacts of strong ENSO events and TRF on the probability of extreme precipitation: Model (b).
**Figure S5**: Synergistic impacts of strong ENSO events and TRF on the probability of extreme temperature: Model (a), produced using the ENSO3.4 index.
**Figure S6**: Synergistic impacts of strong ENSO events and TRF on the probability of extreme precipitation: Model (a), produced using the ENSO3.4 index.
**Figure S7**: Synergistic impacts of strong ENSO events and TRF on the probability of extreme temperature: Model (a), including NPO as part of the group of control variables.
**Figure S8**: Synergistic impacts of strong ENSO events and TRF on the probability of extreme temperature: Model (a), including AO as part of the group of control variables.
**Figure S9**: Hotspots with high population exposure and changes in extreme temperature risk due to ENSO−TRF interactions.
**Figure S10**: Hotspots with high population exposure and changes in extreme precipitation risk due to ENSO−TRF interactions.
**Figure S11**: Hotspots with high GDP exposure and changes in extreme temperature risk due to ENSO−TRF interactions.
**Figure S12**: Hotspots with high GDP exposure and changes in extreme precipitation risk due to ENSO−TRF interactions.

## Data Availability

The radiative forcing and climate variability modes time series used in this paper are available at Figshare. The extreme temperature and precipitation indices are available at https://www.metoffice.gov.uk/hadobs/hadex3/. The CLIMRISK socioeconomic datasets are available at https://datapincc.unam.mx/datapincc/#.
