# Supplementary material for: Synergies Between Observed Warming and ENSO Episodes on Extreme Events
Source: Ann N Y Acad Sci. 2025 Nov 4;1554(1):95–109. doi: 10.1111/nyas.70122 (PMC12728335; doi:10.1111/nyas.70122)
Supplement: Supplementary file 1 — Figure S1: Significance of the ENSO effects on the probability of extreme temperature: Model (a). Figure S2: Significance of the ENSO effects on the probability of extreme precipitation: Model (a). Figure S3: Synergistic impacts of strong ENSO events and TRF on the probability of extreme temperature: Model (b). Figure S4: Synergistic impacts of strong ENSO events and TRF on the probability of extreme precipitation: Model (b). Figure S5: Synergistic impacts of strong ENSO events and TRF on the probability of extreme temperature: Model (a), produced using the ENSO3.4 index. Figure S6: Synergistic impacts of strong ENSO events and TRF on the probability of extreme precipitation: Model (a), produced using the ENSO3.4 index. Figure S7: Synergistic impacts of strong ENSO events and TRF on the probability of extreme temperature: Model (a), including NPO as part of the group of control variables. Figure S8: Synergistic impacts of strong ENSO events and TRF on the probability of extreme temperature: Model (a), including AO as part of the group of control variables. Figure S9: Hotspots with high population exposure and changes in extreme temperature risk due to ENSO−TRF interactions. Figure S10: Hotspots with high population exposure and changes in extreme precipitation risk due to ENSO−TRF interactions. Figure S11: Hotspots with high GDP exposure and changes in extreme temperature risk due to ENSO−TRF interactions. Figure S12: Hotspots with high GDP exposure and changes in extreme precipitation risk due to ENSO−TRF interactions. [file NYAS-1554-95-s001.docx]

**Synergies between current and future warming levels and ENSO episodes on extreme events**

Francisco Estrada^1,2,3*^, Pierre Perron^4^ & Yohei Yamamoto^5,6^

*^1^Instituto de Ciencias de la Atmósfera y Cambio Climático, Universidad Nacional Autónoma de México, Ciudad Universitaria, Circuito Exterior, 04510 Mexico, DF, Mexico, ^2^Institute for Environmental Studies, Vrije Universiteit, Amsterdam, Netherlands, ^3^Programa de Investigación en Cambio Climático, Universidad Nacional Autónoma de México, Ciudad Universitaria, Circuito Exterior, 04510 Mexico, DF, Mexico, ^4^Department of Economics, Boston University, 270 Bay State Rd., Boston, MA, 02215, USA. ^5^Department of Economics, Hitotsubashi University, 2-1 Naka, Kunitachi, Tokyo, Japan, 186-8601, ^6^Tokyo Tech Academy of Energy and Informatics, Tokyo Institute of Technology, Tokyo, Japan.*

***Supplementary Information***

***Supplementary Figures.***

**Figure S1. Significance of the ENSO effects on the probability of extreme temperature: Model a.** See the Methods section for a detailed description of how these significance tests were conducted.

**Figure S2. Significance of the ENSO effects on the probability of extreme precipitation: Model a.** See the Methods section for a detailed description of how these significance tests were conducted.

**Figure S3. Synergistic Impacts of Strong ENSO Events and TRF on the Probability of Extreme Temperature: Model b.** A strong El Niño is defined as a scenario in which the SOI continues to be -2 standard deviations of the historical SOI series (1901:1-2018:12) over the current and the past two quarters consecutively. A strong La Niña is defined similarly, but the SOI index is +2 standard deviations. The synergistic impact of these events and the TRF is assessed across each geographical grid by calculating the effect of a strong ENSO event on the estimated probability of exceeding the extreme threshold under the 2018 TRF level and that calculated under a preindustrial TRF level. Here, the threshold is set at the 90th percentile of the highest daily temperatures in the three-month period of a calendar year from 1961 to 1990. The synergistic effects in the left panels are denoted by $\lambda_{i,ElNiño}$and those in the right panels are denoted by $\lambda_{i,LaNiña}$ as detailed in Supplementary Methods.

**Figure S4. Synergistic Impacts of Strong ENSO Events and TRF on the Probability of Extreme Precipitation: Model b.** A strong El Niño is defined as a scenario in which the SOI remains to be -2 standard deviations of the historical SOI series (1901:1-2018:12) over the current and the past two quarters consecutively. A strong La Niña is defined similarly, but the SOI index is +2 standard deviations for the same duration. The synergistic impact of these events and the TRF is assessed across each geographical grid by calculating the effect of a strong ENSO event on the estimated probability of exceeding the extreme threshold under the 2018 TRF level and that calculated under a preindustrial TRF level. Here, the threshold is set at the 90th percentile of the largest daily precipitations in the three-months period of a calendar year from 1961 to 1990. The synergistic effects in the left panels are denoted by $\lambda_{i,ElNiño}$and those in the right panels are denoted by $\lambda_{i,LaNiña}$ as detailed in Supplementary Methods.

**Figure S5. Synergistic Impacts of Strong ENSO Events and TRF on the Probability of Extreme Temperature: Model a, produced using the ENSO3.4 index.** A strong El Niño is defined as a scenario in which the ENSO3.4 continues to be +2 standard deviations of the historical ENSO3.4 series (1901:1-2018:12) over the current and the past two quarters consecutively. A strong La Niña is defined similarly, but the ENSO3.4 index is -2 standard deviations. The synergistic impact of these events and the TRF is assessed across each geographical grid by calculating the effect of a strong ENSO event on the estimated probability of exceeding the extreme threshold under the 2018 TRF level and that calculated under a preindustrial TRF level. Here, the threshold is set at the 90th percentile of the highest daily temperatures in the three-month period of a calendar year from 1961 to 1990. The synergistic effects in the left panels are denoted by $\lambda_{i,ElNiño}$and those in the right panels are denoted by $\lambda_{i,LaNiña}$ as detailed in Supplementary Methods.

**Figure S6. Synergistic Impacts of Strong ENSO Events and TRF on the Probability of Extreme Precipitation: Model a, produced using the ENSO3.4 index.** A strong El Niño is defined as a scenario in which the ENSO3.4 remains to be +2 standard deviations of the historical ENSO3.4 series (1901:1-2018:12) over the current and the past two quarters consecutively. A strong La Niña is defined similarly, but the ENSO3.4 index is -2 standard deviations for the same duration. The synergistic impact of these events and the TRF is assessed across each geographical grid by calculating the effect of a strong ENSO event on the estimated probability of exceeding the extreme threshold under the 2018 TRF level and that calculated under a preindustrial TRF level. Here, the threshold is set at the 90th percentile of the largest daily precipitations in the three-months period of a calendar year from 1961 to 1990. The synergistic effects in the left panels are denoted by $\lambda_{i,ElNiño}$and those in the right panels are denoted by $\lambda_{i,LaNiña}$ as detailed in Supplementary Methods.

**Figure S7. Synergistic Impacts of Strong ENSO Events and TRF on the Probability of Extreme Temperature: Model a including NPO as part of the group of control variables.**


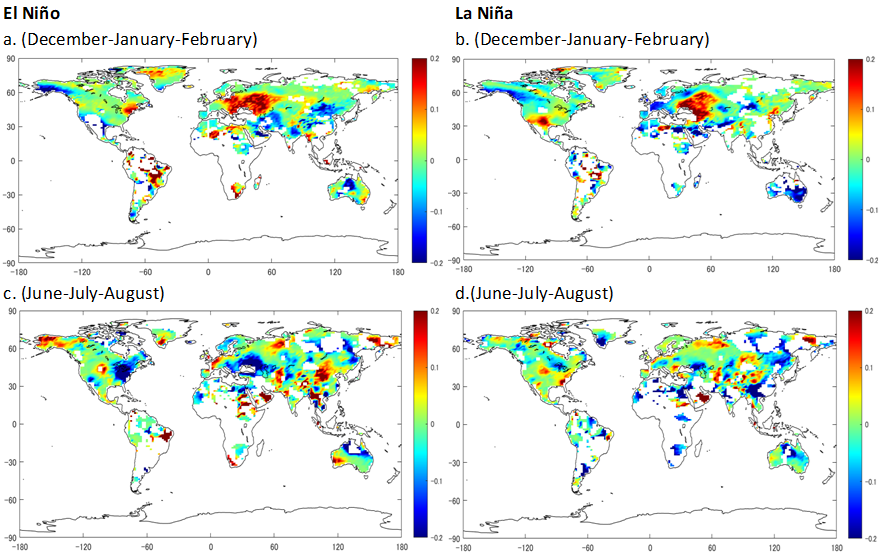


**Figure S8. Synergistic Impacts of Strong ENSO Events and TRF on the Probability of Extreme Temperature: Model a including AO as part of the group of control variables.**

**Figure S9. Hotspots with high population exposure and changes in extreme temperature risk due to ENSO-TRF interactions**. Bubbles are displayed on grid cells with high population levels. The bubble size indicates the population exposed within the grid, while the color illustrates changes in the synergistic risk of extreme temperature for El Niño-TRF in the left panels and those for La Niña-TRF in the right panels: light green signifies decreases in risk, while yellow, orange, and red denote moderate (0%-5%), high (5%-15%) and extreme (>15%) increases in risk, respectively.

**Figure S10. Hotspots with high population exposure and changes in extreme precipitation risk due to ENSO-TRF interactions**. Bubbles are displayed on grid cells with high population levels. The bubble size indicates the population exposed within the grid, while the color illustrates changes in the synergistic risk of extreme precipitation for El Niño-TRF in the left panels and those for La Niña-TRF in the right panels: light green signifies decreases in risk, while yellow, orange, and red denote moderate (0%-5%), high (5%-15%) and extreme (>15%) increases in risk, respectively.

**Figure S11. Hotspots with high GDP exposure and changes in extreme temperature risk due to ENSO-TRF interactions**. Bubbles are displayed on grid cells with high GDP levels. The bubble size indicates the GDP exposed within the grid, while the color illustrates changes in the synergistic risk of extreme temperature for El Niño-TRF in the left panels and those for La Niña-TRF in the right panels: light green signifies decreases in risk, while yellow, orange, and red denote moderate (0%-5%), high (5%-15%) and extreme (>15%) increases in risk, respectively.

**Figure S12. Hotspots with high GDP exposure and changes in extreme precipitation risk due to ENSO-TRF interactions**. Bubbles are displayed on grid cells with high GDP levels. The bubble size indicates the GDP exposed within the grid, while the color illustrates changes in the synergistic risk of extreme precipitation for El Niño-TRF in the left panels and those for La Niña-TRF in the right panels: light green signifies decreases in risk, while yellow, orange, and red denote moderate (0%-5%), high (5%-15%) and extreme (>15%) increases in risk, respectively.
